# Immunotherapeutic approaches for hepatocellular carcinoma

**DOI:** 10.18632/oncotarget.15406

**Published:** 2017-02-16

**Authors:** Vito Longo, Antonio Gnoni, Andrea Casadei Gardini, Salvatore Pisconti, Antonella Licchetta, Mario Scartozzi, Riccardo Memeo, Vincenzo Ostilio Palmieri, Giuseppe Aprile, Daniele Santini, Patrizia Nardulli, Nicola Silvestris, Oronzo Brunetti

**Affiliations:** ^1^ Medical Oncology Unit, Hospital of Taranto, Taranto, Italy; ^2^ Medical Oncology Unit, Hospital of Gallipoli, Gallipoli, Italy; ^3^ Department of Medical Oncology, Istituto Scientifico Romagnolo per lo Studio e la Cura dei Tumori IRCCS, Meldola, FC, Italy; ^4^ Department of Medical Oncology, University of Cagliari, Cagliari, Monserrato, CA, Italy; ^5^ Department of Hepatobiliary Surgery, Ospedale Regionale “F.Miulli”, Strada Pr. Acquaviva - Santeramo, Bari, Italy; ^6^ Department of Biomedical Sciences and Human Oncology, Clinica Medica “A. Murri”, University of Bari Medical School, Bari, Italy; ^7^ Department of Oncology, San Bortolo Hospital ULSS 6, Vicenza, Italy; ^8^ Medical Oncology Unit, University Campus Biomedico, Rome, Italy; ^9^ Pharmacy Unit, National Cancer Research Centre, Istituto Tumori Giovanni Paolo II, Bari, Italy; ^10^ Medical Oncology Unit, National Cancer Research Centre, Istituto Tumori Giovanni Paolo II, Viale Orazio Flacco, Bari, Italy

**Keywords:** adoptive immunotherapy, dendritic cell vaccination, hepatocellular carcinoma, immunotherapy, immune checkpoint

## Abstract

Hepatocellular carcinoma (HCC) is a cancer with a high mortality rate due to the fact that the diagnosis usually occurs at anadvanced stage. Even in case of curative surgical treatment, recurrence is common. Sorafenib and regorafenib are the only therapeutic agents that have been demonstrated to be effective in advanced HCC, thus novel curative approaches are urgently needed. Recent studies focus on the role of immune system in HCC. In fact, the unique immune response in the liver favors tolerance, which can represent a real challenge for conventional immunotherapy in these patients. Spontaneous immune responses against tumor antigens have been detected, and new immune therapies are under investigation: dendritic cell vaccination, immune-modulator strategy, and immune checkpoint inhibition. In recent years different clinical trials examining the use of immunotherapy to treat HCC have been conducted with initial promising results. This review article will summarize the literature data concerning the potential immunotherapeutic approaches in HCC patients.

## INTRODUCTION

Hepatocellular carcinoma (HCC) is one of most frequently diagnosed malignances and the third cause of cancer-related deaths worldwide, with about 600,000 death/years [1]. About 80%-90% of HCC developed into cirrhotic liver disease due to chronic viral hepatitis B or C [2]. Although surgery plays a fundamental role in the early stages, most of HCC patients diagnosed at an advanced stage of the disease or with hepatic impairment are treated with loco-regional or systemic therapies. Recurrence is observed in most of them within 5 years [3]. Only sorafenib and regorafenib, two oral multi-kinase inhibitors, have shown efficacy in advanced stages of this disease, providing a short increase of median overall survival (mOS) [4–6].

Gastrointestinal cancer immunotherapy has had significant progress in the last few years [7]. In particular, recent studies focus on the role of immune system in HCC. The unique immune response in the liver favors tolerance, which could represent a genuine challenge for conventional therapies in HCC patients [8].

## FROM LIVER IMMUNE SYSTEM TO HCC IMMUNE DISORDERS AND IMMUNOTHERAPEUTIC STRATEGY

The liver is an organ with a specific blood supply. Approximately 25% and 75% of the blood enters the liver through the hepatic artery and the portal vein, respectively. The latter drains into smaller diameter structures called sinusoids. Vascular resistance is very low in these structures, and the portal venous blood, which is loaded with nutrients and many microbial antigens from the intestine, flows extremely slowly into the sinusoids. In this phase, antigens are in contact with a great number of non-parenchymal cells including liver sinusoidal endothelial cells (LSECs), hepatic stellate cells (h-SCs), Kupffer cells, dendritic cells (DCs), and lymphocytes. All these actors are potential protagonists in immune response.

**Figure 1:**
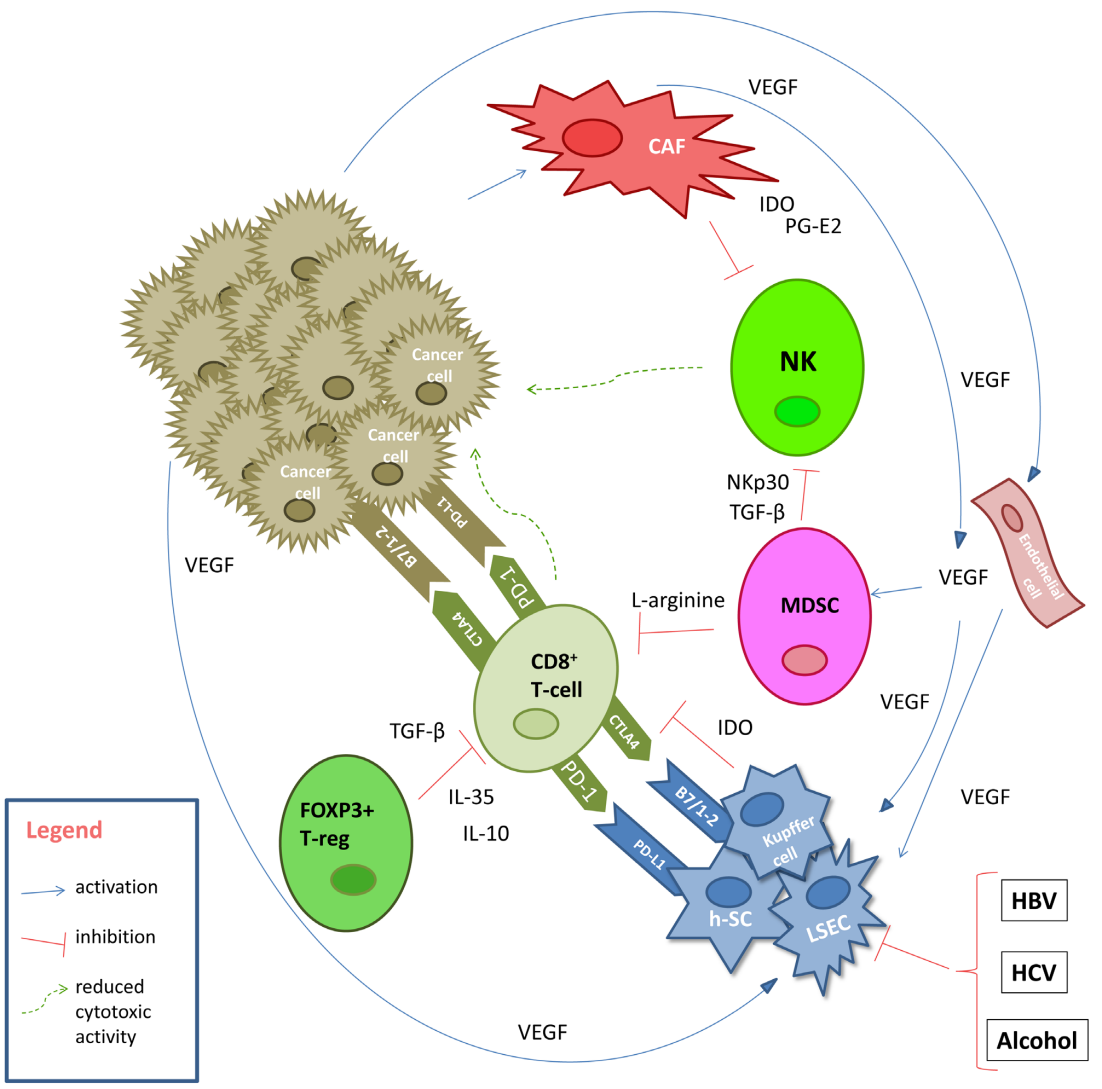
Pathogenetic mechanisms of immune-tolerance in HCC Abbreviation - CAF: cancer associated fibroblast; CTLA4: cytotoxic T-limphocite antigen-4; HBV: hepatits B virus; HCV: hepatits C virus; h-SC: hepatic stellate cells; IDO: indoleamine 2,3 dioxygenase; IL: interleukin; LSEC: liver sinusoidal endothelial cells; MDSC: myeloid-derived suppressor cells; NK: natural killer; NKp30: natural killer protein 30; PD-1:programmed cell death protein 1; PD-L1: programmed cell death protein ligand 1; PG-E2: prostaglandin E2; TGF-β: transforming growth factor β; VEGF: vascular endothelial growth factor.

Under physiological conditions, different digestion metabolites and bacteria coming from the bowel to the liver passing through the portal system represent an antigenic hyperstimulation. To overcome autoimmune mechanisms, the liver develops a series of mechanisms aiming at self-tolerance: i) decrease of costimulatory immune receptors such as B7-1, B7-2; ii) up-regulation of programmed cell death protein 1 (PD-1) receptor and cytotoxic T-limphocite antigen-4 (CTLA4) immuno-checkpoint inibitors on hepatic antigen presenting cells (h-APC) such as h-SCs, LSECs, and Kupffer cells [9,10]; iii) secretion of cytokines with interleukin (IL)-10 and transforming growth factor β (TGF-β) most studied [11,12]. Similarly, hepatitis B virus or hepatitis C virus (HCV) infection, autoimmune hepatitis, alcohol abuse, non-alcoholic steatohepatitis lead to frequent chronic inflammatory liver insult resulting in a deregulation of T cell activities [13] with an increase of the expression of immune checkpoint inhibitors on h-APCs [14]. Moreover, tumor growth is favored by these mechanisms, which result amplified in HCC patients [15]. Cancer associated fibroblasts, essential components of the HCC microenviroment, inhibit natural killer (NK) cell function by releasing the immunosuppressive molecules prostaglandin E2 and indoleamine 2,3 dioxygenase (IDO) [16]. Moreover, several data support a possible correlation between the phenotype of infiltrating lymphocytes and the risk of relapse after transplantation or loco-regional treatments [17]. With regard to this, the expression of T-helper1 cytokines such as IL-1α, IL-1β, IL-2, and interferon (IFN)-γ in tumor tissue is associated with good prognosis, whereas T-helper2 cytokines such as IL-4, IL-5, and IL-10 are upregulated in aggressive disease [8]. As well as in other solid tumors, forkhead box P3 (FOXP3) + T-regs, a subset of CD4+ T cells specialized in the suppression of the host immune system against self antigens, promotes tumor development inducing a state of severe immune-suppression in HCC [18]. The suppressive function of FOXP3+ T-regs may be related to different mechanisms such as target cells killing by T-regs, modulation of target cell signaling via cell-cell contact, and secretion of immunosuppressive cytokines such as IL-10, IL-35, and TGF-β. The epigenetic regulation is patho-physiologically relevant for T-regs function and development. Furthermore, epigenetic mechanisms responsible for regulating the foxp3 gene expression have a key role in T-regs suppressive activity [19,20]. Moreover, an increased number of CD4^+^CD25^+^FOXP3^+^ Treg may reduce the activity of CD8^+^ T cells, promoting disease progression, with high mortality and reduced survival of these patients [21]. Subsequently, programmed cell death protein-ligand (PD-L) expression and FOXP3+ T-reg cell expression have been analyzed on tissue of 240 resected HCC patients, with a cross validation in an independent cohort of 125 HCC samples. Patients with a higher PD-L1 tumor expression had a significantly poorer prognosis than patients with lower expression. The multivariate analysis demonstrated that PD-L1 expression was an independent predictor of recurrence after surgery. The prognostic value of PD-L1 expression was validated in the independent data set. PD-Ls expression significantly correlated with FOXP3+ lymphocyte infiltration. Moreover, tumor-infiltrating PD1^+^CD8^+^ cytotoxic cells and T-reg cells were also independent prognostic factorsfor overall survival (OS) and post operative recurrence. [22]. However, it should be considered that, until today, the biological bases of pathogenesis/progression of HCC remain poorly defined. Figure 1 describes the principal pathogenetic mechanisms of immune-tolerance involved in HCC.

## INTERACTION BETWEEN ANTI-VEGF THERAPY AND HCC IMMUNOSURVEILLANC

Vascular endothelial growth factor (VEGF)/VEGF receptor (VEGFR) axis, one of the most important molecular pathways controlling angiogenesis, plays a key role in tumor microenviroment including immunosupression. VEGF is known to inhibit DCs maturation through the activation of NF-KB which is able to influence the differentiation of monocytes into DCs. In addition, VEGF promotes an immunosuppressive phenotype both increasing the DCs production of IDO, a strong inhibitor of T-cell activation, as well as inactivating STAT3 [23]. Interestingly, Motz et al. reported that VEGF could induce Fas Ligand expression in tumor endothelial cells, inhibiting cytotoxic T-lymphocyte (CTL) infiltration in the tumor and reducing their cytotoxic activity [24]. Voron et al., on the other hand, showed that VEGF induces exhaustion in intratumoral CD8+ T cells by promoting the expression of PD-1 and other checkpoint molecules such us CTLA-4 and TIM-3 [25]. Myeloid-derived suppressor cells (MDSCs) were shown to inhibit NK cell activation though TGF-β and NK protein 30 and to regulate T-cell proliferation by L-arginine. Intratumoral MDSCs infiltration correlates with circulating VEGF levels in several solid tumors, including HCC [26]. Furthermore, injection of recombinant VEGF increased MDSC population *in vivo*, and liver-specific overexpression of VEGF led to accumulation of proangiogenic MDSCs in a SDF1α/CXCR4 pathway-dependent manner [27].

In line with these data, anti-VEGF treatment may have potential immune-modulatory functions. Sorafenib, the first therapy approved in advanced HCC stage, exerts its activity by a pan-VEGF receptor inhibition. Preclinical studies in liver cancer models showed that sorafenib decreases MDSC and T-reg levels in spleen, bone marrow and tumor, restraining their function as well [28]. Nevertheless, sorafenib seems to decrease IL-12 expression and to impair DCs function [29]. At the same time, excessive pruning of tumor vasculature over time could aggravate hypoxia in the tumor microenviroment, resulting in high hypoxia-inducible factor-1α (HIF)-1α levels, which enhances immune checkpoint molecules expression such as PD-L1 in MDSCs and macrophages [30].

Chen et al. examined the effects of implementing anti-PD1 monoclonal antibody (mAb) to sorafenib in mice with HCC. The increased hypoxia after sorafenib resulted in increased intratumoral levels of PDL-1 and up-regulation of SDF1α/CXCR-4 pathway with a consequent accumulation of immunosuppressive cells. Anti-PD-1 mAb administration did not significantly delay tumor growth when combined with sorafenib alone, even if the combination of an anti-PD-1 with sorafenib and AMD3100, an anti-CXCR-4 molecule, notably restrained tumor growth and metastasis [31].

According to these results, a potential future approach could be represented by a careful titration of VEGF inhibition with the aim to block the VEGF pathway and contemporarily alleviate hypoxia by vascular normalization, enhancing immunotherapy efficacy [32]. Trials evaluating a combined approach comprising anti-angiogenesis drugs and immunotherapy are currently ongoing (Table 1).

**Table 1:** Combination of anti-angiogenetictherapy and immunotherapy clinical trials

Phase	Anti-angiogenesis drug	Immunecheckpoints blocker	Design	Primary endpoint(s)	Clinical trail ID
I	Angiokinase inhibitor targeting VEGFR 1-3, FGFR 1-3, and PDGFR Α/Β (*nintedanib*)	IGG4 anti-PD-1 blocking mAb (*pembrolizumab*)	Pembrolizumab + nintedanib (PEMBIB) in second line HCC	Maximum tolerated dose and dose limiting toxicities	NCT02856425
I	Anti-VEGFR2 antibody (*ramucirumab*)	Anti–PD-L1 immune checkpoint inhibitor (*MEDI4736*)	Ramucirumab + MEDI4736 in metastatic or locally advanced and unresectable HCC	Dose limiting toxicities	NCT02572687
I/II	VEGFR2-TKI (*apatinib*)	Anti-PD-1 mAb (*SHR-1210*)	Apatinib + SHR-1210 in advanced HCC	Overall survival rate	NCT02942329
III	VEGFR –TKI (sorafenib)	Pexastimogene devacirepvec (*Pexa-Vec*)	Sorafenib VS sorafenib + Pexa-Vac in advanced HCC	Overall survival	NCT02562755

Abbreviation – FGFR: fibroblast growth factor receptor; HCC: hepatocellular carcinoma; mAb: monoclonal antibody; PDGFR: platlet derived growth factor receptor; TKI: tyrosin kinase inhibitor; VEGFR: vascular endothelial growth factor receptor.

## HCC IMMUNE-RESPONSE STRATEGIES

**Figure 2:**
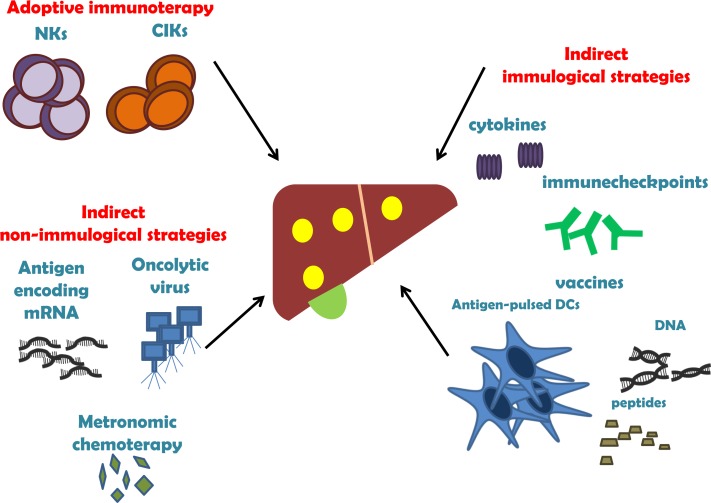
The three main strategies of HCC immunotherapy

There are three main strategies to improve tumor-specific immune response (Figure 2): i) *adoptive immunotherapy* - HCC-epitopes immunized cells, which recognize and act against cancer cells; ii) *indirect immunological strategies* - cytokines, immune checkpoint blockade mAbs, cancer vaccines used to increase immune system activity; iii) *indirect non immunological strategies* - antigen-encoding mRNA strategy in HCC, metronomic chemotherapy, oncolytic viruses [33]. Table 2 summarises the main clinical trials and restrospective studies or meta-analysis in HCC immunotherapy.

**Table 2:** Clinical trials and retrospective studies or meta-analysis in immunotherapy treatments of HCC

Immune-therapy strategies	Trial/study	Design	Results	Ref.
Adoptive immunotherapy	Randomized phase II	Adjuvant 6 versus 3 courses of CIK cell infusion immunotherapy in resected patients	1-, 3-, 5-y - DFS rates 6 courses CIK infusion: 84.7%, 30.5%, 19.4% 3 courses CIK infusion: 83.1%, 31.7%, 23.3%	39
	Retrospective analysis	Adjuvant CIK cell infusion immunotherapy in resected patients	1, 2, 3, 4, 5-y OS rates Untreated group: 84%, 69.2%, 61.6%, 56.9%, 50.2 % CIK cells group: 93.6%, 83.3%, 76.6%, 71.1%, 65.9%	40
	Retrospective analysis	Adjuvant autologous CIK cell infusion immunotherapy versus control after locoregional procedures	Overall RRs: 79.8% *versus* 76.5% OS: 56 months *versus* 31 months PFS: 17 months *versus* 10 months	41
	Phase III	Adjuvant activated CIK cells infusion immunotherapy versus control in resected patients or after RFA or after percutaneous ethanol injection	mDFS: 44 months *versus* 30 months	42
	Meta-analysis including 13 phase II/III trials	Adjuvant activated CIK cells infusion immunotherapy after RFA and/or TACE	1-y OS - OR=0.25 (95% CI, *p* < 0.001) 2-y OS - OR=0.17 (95% CI, *p* < 0.001)	43
Indirect immunological strategies	Randomized phase II	TACE plus IFN-α versus TACE in unresectable HCC	mOS: 29 months *versus* 26 months (*p* = 0.003) mDFS: 23.6 months *versus* 20.3 months (*p* = 0.027)	51
	Phase II	Combined intrarterial 5-FU plus PEG-IFN α-2b in advanced HCC with portal venous invasion	ORR: 73% mOS: 29.9 months	52
	Meta-analysis of 10 trials	Adjuvant IFN versus placebo	Recurrence rate - OR: 0.42 (CI 95%; *p* < 0.00001)	53
	Phase I	Tremelimumab in advanced HCC	Good toxicity profile PR: 17.6% SD: 45%	58
	Phase I/II	Nivolumab in advanced HCC	Good toxicity profile ORR: 20% Median duration of response: 9.9 months	62
	Phase I	Pulsed DCs by autologous cells from tumor lysate in advanced HCC	Positive feasibility Low toxicity profile	70
	Phase II	DCs pulsed with HepG2 cell lysate in advanced HCC	DCR:28% Low toxicity profile	71
	Phase I	AFP-derived vaccine in advanced HCC	T-cell increased activity in all patients	74
	Phase I	GPC3 vaccine advanced HCC	mOS: 12.2 months in high T-cell expressing patients mOS: 8.5 months in low expressing patients	75
	Phase II	Telomerase peptide GV1001 vaccine in advanced HCC	Low toxicity profile 6 mo SD: 45.9% mTTP: 57 days	78
	Phase II	Second line therapy with lenalidomide	OS: 7.6 months PR: 15%	
Indirect non immunological strategies	Phase II	Metronomic capecitabine in previously untreated and resistant to/intolerant of sorafenib advanced HCC	mPFS (untreated): 6 months mPFS (second line): 14.4 months	89
	Randomized phase II	Two doses of JX-594 (high versus low dose) vaccinia virus in advanced HCC	Positive feasibility mOS: 14.1 *versus* 6.7 months	90

Abbreviation - CI: confidence interval; CIK: cytokine induced killer; DC: dendritic cell; DCR: disease control rate; DFS: disease free survival; HCC: hepatocellular carcinoma; IFN: interferon; mDFS: median disease free survival; mOS: median overall survival; mTTP: median time to progression; ORR: overall response rate; OS: overall survival; PFS: progression free survival; PR: partial response; RFA: radiofrequency ablation; RR: recurrence rate; SD: stable disease; TACE: transarterial chemoembolization.

## ADOPTIVE IMMUNOTHERAPY

Adoptive immunotherapy utilizes NK and cytokine induced killer (CIK) cells for autologous cells reinfusion immunized with cancer epitopes. NK cells are immune lymphocytic cells resident in reticuloendothelial organs with a key role in immune and anti-tumor defense [34].

Thirty-seven HCC patients treated with radiofrequency ablation (RFA) showed a phenotypic and functional activation of autologous NK-cells [35]. Currently, adjuvant autologous NK cell reinfusion is being evaluated in 2 ongoing clinical studies in transplant and resected patients (NCT01147380 [36] and NCT02008929 [37]).

CIK cells are a heterogeneous cell population comprising CD3+ CD56+, CD3+ CD56− and CD3− CD56+ cells, which are able to both recognize tumor antigens and kill cancer cells directly [38]. Several studies reported an improved outcome, using CIK cells as adjuvant therapy after liver surgery in HCC patients. In particular, a phase II study evaluating the adjuvant role of CIK cells considered 127 HCC radically resected patients which were randomized into the following 3 arms: 3 or 6 courses of adjuvant CIK cells infusion immunotherapy or observation only. Disease free survival (DFS) rate at 1, 3, and 5-year was 83.1%, 31.7%, and 23.3% in 3 courses CIK cell infusion, 84.7%, 30.5%, and, 19.4% in 6 courses CIK cell infusion, and 82.6%, 20.9%, and 11.2% in the control group, respectively. A statistically significant difference between 3 courses CIK cells (*p* = 0.001) and 6 courses CIK cells (*p* = 0.004) as compared to the control group was observed in the absence of a statistically difference between CIK cell groups [39]. Similarly, a large retrospective study comparing surgery alone (206 patients) with surgery followed by CIK cell transfusion (204 patients) showed a significantly better prognosis in the CIK group. The 1-, 2-, 3-, 4-, and 5-year OS rates of CIK group were higher than surgery alone group: 93.6%, 83.3%, 76.6%, 71.1%, 65.9% and 84%, 69.2%, 61.6%, 56.9%, 50.2%, respectively, with a significant difference between the two groups (log-rank test; *p* = 0.0007). Moreover, patients treated with more than 8 cycles of cell transfusion showed significantly better survival than those treated with less than 8 cycles (*p* = 0.0272). CIK group displayed significantly better OS than surgery-alone group in patients with more than 5-cm tumors (p = 0.0002) [40].

Another retrospective study compared 85 patients treated with adoptive autologous CIK cell transfusion in combination with transarterial chemoembolization (TACE) plus RFA, versus 89 patients treated with loco-regional therapy alone. Despite similar response rates, the TACE+RFA+CIK cell group showed a significant improvement in terms of longer progression free survival (PFS) (17 months versus 10 months, *p* = 0.001) and OS (56 months versus 31 months, *p* = 0.001) compared to the TACE+RFA group [41]. Recently, in a multicenter phase III study, 230 HCC patients treated by surgery, RFA or percutaneous ethanol injection were randomized to receive adjuvant autologous CIK cells infusion or not, showing a median DFS of 44 months and 30 months, respectively (p = 0.010 by 1-sided log-rank test) [42]. A meta-analysis, including 13 phase II/III trials evaluating the use of the CIK cell adjuvant after RFA and TACE, demonstrated a significantly improved 1-year OS (OR = 0.25, 95% CI: 0.12 to 0.52, *p* < 0.001) and 2-year OS (OR = 0.17, 95% CI: 0.07 to 0.43, *p* < 0.001) [43]. Finally, a systematic review of the literature, including 14 eligible articles, confirmed that CIK cells could prevent recurrence in resected HCC [44]. Currently, several phase III/IV studies are ongoing on autologous CIK cell infusion adjuvant therapy. NCT01749865 [45] and NCT00769106 [46] are two phase III studies which will assess time to recurrence after surgery as the primary end-point in HCC patients who underwent to radical resection.

In conclusion, the adoptive immunotherapy achieved favorable results in particular in the adjuvant setting leading to these last phase III trials in HCC.

## INDIRECT IMMUNOLOGICAL STRATEGIES

### Interferons

Recombinant human IFN-α was the first immunotherapy to undergo substantial clinical development in HCC, due to the extensive experience gained in the treatment of chronic viral hepatitis as well as to its anti-angiogenic and immunostimolatory proprieties [47]. *In vitro* studies showed a pro-apoptotic effect of IFN-α, IFN-β, and IFN-γ on HCC cells [48–50]. A phase II randomized trial comparing TACE plus IFN-α versus TACE in unresectable HCC showed median DFS of 23.6 months and 20.3 months in TACE-IFN-α and TACE groups, respectively (*p* = 0.002). mOS of 29 months and 26 months were observed in the TACE-IFN-α and control group, respectively (*p* = 0.003) [51]. A phase II trial evaluating the efficacy of combined intrarterial 5-fluorouracil (5-FU) and systemic pegylated IFN α-2b in patients with advanced HCC with portal venous invasion reported mOS of 29.9 months [52]. A meta-analysis of 10 trials (8 randomized and 2 non-randomized controlled studies) demonstrated that recurrence rate (RR) was significantly lower in patients treated with adjuvant IFN with respect to the placebo group (OR = 0.66; 95% CI = 0.50 to 0.86; *p* = 0.02). Death rates were significantly decreased (OR = 0.42; 95% CI = 0.32 to 0.56; *p* < 0.00001) and subgroup analysis showed an advantage for the group treated with TACE (OR = 0.33; 95% CI = 0.21 to 0.50; *p* < 0.00001) compared to patients treated with surgery (OR = 0.51; 95% CI = 0.36 to 0.72; *p* = 0.0002) [53]. An *in vivo* study demonstrated that sorafenib in combination with IFN synergistically suppressed tumor growth, inducing apoptosis [54]. An ongoing, phase II randomized trial is comparing IFN-α plushepatic arterial infusion 5-FU versus cisplatin plus 5-FU in HCC patients after liver resection (NCT01834963) [55].

### Immune checkpoint inhibitors

Several negative immunologic regulator targets revolutionized cancer care in melanoma, renal cancer, and non small cell lung cancer with significant improvement in terms of mOS and response rate [56]. CTLA-4 and PD-1 are the most studied immune checkpoint inhibitors in HCC [57].

Tremelimumab, an anti-CTLA-4 mAb, has been tested in a phase I trial recruiting 21 advanced HCC patients. This molecule was well tolerated with 17.6% and 58.8% of partial response (PR) and stable disease, respectively. Interestingly, an over 200-fold decrease of serum viral load in 12 HCC HCV infected patients treated with tremelimumab has been seen [58]. A phase I trial of tremelimumab in combination with RFA or TACE has been concluded (NCT01853618) [59]. This combined therapy proved to be safe and of the 10 patients evaluable for response outside loco-regional treatments, all showed immune cell infiltration and 4 achieved confirmed PR [60].

Nivolumab, a fully human mAb anti-PD-1 has been tested in advanced HCC patients in the CheckMate 040 study. It presented a fairly good toxicity profile with a 20% of response rate in 214 patients treated in the dose expansion phase. Median duration of response was 9.9 months with a disease control rate (DCR) of 64%. Moreover, 9-months OS rate in the expansion phase was 74% (95% CI 67-79) [61].

Preliminary studies evaluating the role of MEDI4736, an anti PD-L1 mAb, suggested a favoreable toxicity profile with a DCR at 12 weeks of 21% [62].

The study protocols of two clinical trials involving the anti-PD-1 mAb pembrolizumab have been recently presented at ASCO 2017 Gastrointestinal Cancers Symposium. The former is a single-arm, multicentric, phase 2 study (KEYNOTE-224) designed to evaluate the efficacy and safety of pembrolizumab in previously treated advanced HCC patients. [63]. The latter is a double-blind, placebo-controlled phase 3 study (KEYNOTE-240) which will randomize pembrolizumab versus placebo in previously treated advanced HCC [64].

In a murine melanoma vaccine model, inhibition of both CTLA-4 or PD-1 increased the proportion of CTLA-4 and PD-1-expressing CD4/CD8 tumor infiltrating T effector cells and decreased intratumoral T regulatory cells, as compared to either agent alone [65]. Given the increased efficacy observed with combination approaches in other tumor types, the Checkmate 040 trial will evaluate the efficacy and toxicity of the combination of ipilimumab plus nivolumab (NCT01658878) [66].

Currently, several anti-immunocheckpoint inhibitor mAbs are being developed (Table 3). These drugs are radically changing the approach to oncological diseases, being able to offer both a longer duration of response and a better toxicity profile compared to chemotherapy or target therapy. Nevertheless, the economic impact of these drugs will force clinicians and researchers to select patients who should be elegible for these treatments. In this scenario, the identification of biomarkers able to predict the response to checkpoint blockades represents an intriguing area of research. Immunohistochemical evaluation of PD-L1 is a tested predictive biomarker for the response to PD-1/PD-L1 mAbs. Indeed, the histological detection of tumor infiltration of immune cells or their molecules in the tumor microenvironment may be indirect predictive biomarkers of response to PD-1/PD-L1 checkpoint blockades. Moreover, mismatch-repair deficiency gene analysis should improve the clinical benefit of immune checkpoint inhibitors [67].

**Table 3:** Clinical trials of immune-checkpoint blocker in HCC patients

Immune-checkpoints target	Drug	Associated treatment	Phase	Status	Results	Clinical trial ID
CTL-A4	Tremelimumab	None	II	Completed	PR: 17.6%; SD: 58.8%	NCT01008358
	Tremelimumab	TACE or ablation	I	Recruiting	mPFS for study population (n = 17): 7.4 months	NCT01853618
PD-L1/CTL-A4	Durvalumab/Tremelimumab	TACE or RFA	I/II	Recruiting	NA	NCT02821754
	Nivolumab/Ipilimumab	None	I/II	Recruiting	NA	NCT01658878
PD-1	Nivolumab	TGFBR1 kinase inhibitor (*galunisertib)*	I/II	Recruiting	NA	NCT02423343
	Nivolumab	None	I/II	Recruiting	Good toxicity profile; response rate: 20%; median duration of response: 9.9 months	CA209-040
	Anti-PD-1 antibody (not specified)	Decitabine	I/II	Recruiting	NA	NCT02961101
	Pembrolizumab	None	II	Recruiting	NA	NCT02702414
	PDR001	c-met inhibitor (INC280)	II	Recruiting	NA	NCT02795429
	PDR001	Anti-TGF beta antibody (NIS793)	II	Not yet recruting	NA	NCT02947165

Abbreviation– CR: complete response; CTLA4: receptor and cytotoxic T-limphocite antigen-4; RFA: radiofrequency ablation; PD-1: programmed cell death protein 1; PD-L1: programmed cell death ligand1; PR: partial response; SD: stable disease; TACE: transarterial chemoembolization.

### Vaccine strategy

The aim of cancer vaccination is the induction and continuance of a tumor-specific immune response by eliciting effector T cells that can specifically decrease tumor load and control tumor relapse. Various approaches have been used in this setting: i) pulsed DCs, ii) peptide-based vaccines, and iii) DNA-based vaccines.

DCs play a key role in both innate and adaptive immunity. They resulted increased in peripheral blood and lymph nodes of HCC patients [68]. Moreover, DC infiltration in HCC lesions has been associated with a better prognosis in resected patients [69]. A phase I trial showed a feasible and well tolerated anti-cancer immunization through the stimulation of DCs by autologous cells from HCC lysate [70]. A phase II trial with DCs pulsed with HepG2 cell lysate demonstrated a DCR of 28 % in the absence of relevant adverse events in 39 advanced HCC patients [71]. Finally, in 20 and 13 HCC patients treated with TACE and TACE followed by DC infusion, respectively, a more effective enhancement of tumor specific immune response with the combination approach was observed, without differences in terms of RR [72]. An ongoing phase I trial is evaluating the administration of intra-tumoral DC allogenic vaccine (NCT01974661) [73]. The external passive immunization of these patients with reinfusion of DCs able to develop an immune response against tumor cells could be a feasible and active strategy even if the complexity of the process may represent a key challenge.

αFP and glypican-3 (GPC3) are the main HCC tumor associated antigens of peptide-based vaccine. αFP-derived vaccine has been evaluated in a phase I trial reporting a T-cell increased specific activity in all patients [74]. Another phase I trial involving 33 advanced HCC patients treated with GPC3 vaccine reported a mOS of 12.2 months (95% CI = 6.5-18.0) and 8.5 months (95% CI = 3.7-13.1) (*p* = 0.033) in patients with a high GPC3-related CTLs expressionand in those with a low GPC3-related CTL expression, respectively [75].

Recently, a promising ongoing phase II trial (UMIN-CTR: 000002614) is evaluating GPC3 vaccine after surgery or RFA [76]. Currently, preclinical data for GPG3 DNA-based vaccines indicated the induction of specific and effective cellular antitumor immunity against GPC3 only in *in vivo* models [77]. No clinical trials are ongoing even if a report of two pretreated αFP positive HCC patients treated with αFP-DNA vaccine and adenovirus driven immunization showed promising safety and immunogenic T cell response [78]. Although it was not a characteristic antigen, the telomerase peptide GV1001 was evaluated in a clinical phase II trial with a median TTP of 57 days [79]. Another therapeutic strategy potentially able to improvethe clinical outcome of these patients could be the combination of vaccination with anti-angiogenic tyrosine-kinase inhibitors (TKIs), such as sorafenib [80]. Regarding this, an ongoing phase III trial is comparing vaccinia virus based immunotherapy plus sorafenib versus sorafenib alone (PHOCUS) (NCT02562755) [81].

Vaccines have been the first therapeutic strategy in immuno-oncology. Anyway, after decades of studies, despite the fact that some positive data are available in literature, there are no phase III trials to prompt this approach in HCC.

### Immunomodulator strategy

Initial data regarding the activity of immunomodulators in HCC treatment are currently avaiable. Lenalidomide has been evaluated in a phase II study after sorafenib failure or intolerance, with PR of 15% and 7.6 months of mOS [82]. In an orthotopic HCC model, lenalidomide plus sorafenib showed an interesting tumor growth inhibition, with a significant increase of T cytotoxic IFN-γ infiltrating tumor cells [83]. Further data are mandatory in this therapeutic setting.

## INDIRECT NON IMMUNOLOGICAL STRATEGIES

### Antigen-encoding mRNA strategy

Another recent vaccination strategy is represented by the antigen-encoding mRNA technique. mRNA encodes genetic information corresponding to whole antigens, with a consequent antigen expression and presentation. This approach is not associated to the risk of genomic integration, with a more favorable safety profile compared to the use of DNA sequences. On one hand, DCs can be cultivated and electroporated with mRNA followed by their restitution into the patient. On the other, “nude mRNA” can be injected intratumorally. In both cases anti-tumour immune responses were achieved in a variety of mouse models [84,85]. Lastly, a recent study is evaluating the role of TriMix mRNA and mRNA encoding the target antigens GPC3 and MAGE-C2 mRNA when injected intranodally the same day as the RFA treatment [86].

### Metronomic chemotherapy

Metronomic chemotherapy is the admnistration of low-dose chemoterapeutic drug for a long period in the absence of drug-freebreaks [87].

Several Authors demonstrated that tumor response to this therapeutic approach is related not only to a direct antineoplastic activity but also to the following immune-stimulatory: i) activation of immunity, ii) induction of tumor dormancy, and iii) chemotherapy-driven dependency of cancer cells [88]. An Italian, phase II non-randomized trial included 59 previously untreated patients with advanced HCC and 31 patients resistant to or intolerant of sorafenib treated with capecitabine 500 mg twice daily until progression of disease or unacceptable toxicity. [89]. Authors reported mPFS of 6 months and 14.4 months in the two cohorts, respectively. Treatment was well tolerated with an acceptable toxicity. Anyway, no immunological analyses have been performed in this trial.

### Oncolytic viruses

Oncolytic viruses have a dual mechanism of action. In fact, they are able to induce both tumor cells lysis during viral replication and unmasking of tumor antigens for cell-mediated activation. A randomized phase II trial tested the feasibility of two doses of JX-594 (Pexa-Vec), an oncolytic and immunotherapeutic vaccinia virus in 30 HCC patients. Treatment was well tolerated with flu-like symptoms, anorexia, lymphopenia, and hypertransaminasemia, and a significantly longer mOS in high-dose arm respect to low-dose arm (14.1 months and 6.7 months, respectively) [90]. On the contrary, JX-594 did not extended mOS with respect to best supportive care in second line therapy after sorafenib [91]. Currently, a phase III study is randomizing the administration of this virus followed by sorafenib versus sorafenib alone in advanced HCC untreated patients (NCT02562755) [81].

## CONCLUSIONS

Today, sorafenib and regorafenib are the only therapies that have shown efficacy in advanced HCC. New molecular approaches have been experimented, without significant improvement of survival. Therefore, we urgently need to identify new therapeutic strategies as well as to select patients suitable for these treatments. Immunotherapy is laying the foundations for solid treatments. In recent years different clinical studies examining the role of immunotherapy to treat HCC have been conducted with initial promising results in particular regarding CIK cells in the adjuvant setting and immune checkpoint inhibitors in advanced stages. The results of the several ongoing trials are warranted. Furthermore, since the hepatic immune system plays an important role in reduction ofthe immune response, the possibility of unmasking these mechanisms seems to be a winning weapon in HCC, with immunotherapy playing a fundamental role in this cancer in the near future.
